# Identification of Latent Sleep Profiles in Middle-Aged Adults and Connections to Well-Being

**DOI:** 10.1093/geroni/igab046.1304

**Published:** 2021-12-17

**Authors:** Claire Smith, Soomi Lee

**Affiliations:** 1 University of South Florida, University of South Florida, Florida, United States; 2 University of South Florida, Tampa, Florida, United States

## Abstract

For middle-aged adults, achieving adequate sleep is a challenge but essential for long-term health. The present study identified latent sleep profiles to clarify how multiple sleep variables (i.e., regularity, satisfaction, alertness, timing, efficiency, and duration) cooccur within middle-aged adults and the implications these holistic sleep experiences have for well-being. Three profiles emerged within the Midlife in the United States II dataset (MIDUS; N=4030, Mage=56.23 years): (i) good sleepers, (ii) nappers/poor night sleepers, and (iii) sufficient but irregular sleepers. Generally, good sleepers reported the best well-being, sufficient/irregular sleepers reported comparatively moderate well-being, and nappers/poor night sleepers reported the worst well-being across a variety of indicators (i.e., chronic health conditions, life satisfaction, positive affect, negative affect, and psychological well-being) after adjusting for sociodemographic characteristics. Age moderated these associations. Our findings advance understanding of sleep health as a multifaceted construct and of its connection to well-being in middle-aged adults.

